# Blue food demand across geographic and temporal scales

**DOI:** 10.1038/s41467-021-25516-4

**Published:** 2021-09-15

**Authors:** Rosamond L. Naylor, Avinash Kishore, U. Rashid Sumaila, Ibrahim Issifu, Blaire P. Hunter, Ben Belton, Simon R. Bush, Ling Cao, Stefan Gelcich, Jessica A. Gephart, Christopher D. Golden, Malin Jonell, J. Zachary Koehn, David C. Little, Shakuntala H. Thilsted, Michelle Tigchelaar, Beatrice Crona

**Affiliations:** 1 Stanford University, Stanford, CA USA; 2 International Food Policy Research Institute (IFPRI), New Delhi, India; 3 University of British Columbia, Vancouver, BC Canada; 4 WorldFish, Bayan Lepas, Malaysia; 5 Michigan State University, East Lansing, MI USA; 6 Wageningen University, Wageningen, The Netherlands; 7 Shanghai Jiao Tong University, Shanghai, China; 8 Pontificia Universidad Católica de Chile, Santiago, Chile; 9 American University, Washington, DC USA; 10 Harvard T.H. Chan School of Public Health, Boston, MA USA; 11 Beijer Institute of Ecological Economics, The Royal Swedish Academy of Sciences, Stockholm, Sweden; 12 Stockholm Resilience Centre, Stockholm University, Stockholm, Sweden; 13 Royal Swedish Academy of Science, Stockholm, Sweden; 14 University of Stirling, Stirling, UK

**Keywords:** Environmental social sciences, Agriculture, Social sciences

## Abstract

Numerous studies have focused on the need to expand production of ‘blue foods’, defined as aquatic foods captured or cultivated in marine and freshwater systems, to meet rising population- and income-driven demand. Here we analyze the roles of economic, demographic, and geographic factors and preferences in shaping blue food demand, using secondary data from FAO and The World Bank, parameters from published models, and case studies at national to sub-national scales. Our results show a weak cross-sectional relationship between per capita income and consumption globally when using an aggregate fish metric. Disaggregation by fish species group reveals distinct geographic patterns; for example, high consumption of freshwater fish in China and pelagic fish in Ghana and Peru where these fish are widely available, affordable, and traditionally eaten. We project a near doubling of global fish demand by mid-century assuming continued growth in aquaculture production and constant real prices for fish. Our study concludes that nutritional and environmental consequences of rising demand will depend on substitution among fish groups and other animal source foods in national diets.

## Abstract (web summary)

Global demand for “blue food” is growing. In this quantitative synthesis, the authors analyse global seafood demand and project trends to 2050, finding considerable regional variation in the relationship between wealth and consumption.

## Introduction

Understanding the demand for aquatic foods is critical for assessing their current and future role in global food systems. A common view is that the production of aquatic foods, referred to here as “blue foods” captured from or cultivated in marine and freshwater systems, will need to expand in coming decades to meet population- and income-driven demand. The regional and species-specific aspects of demand are often obscured, however, raising questions about the alignment of demand and supply across a diverse array of aquatic food systems. This paper examines blue food demand for multiple species groups across regions over time. Unlike other papers that present comprehensive models of fish demand and supply^1–5^, this study provides a synthetic analysis based on secondary data from FAO and The World Bank, parameters from published models, and case studies at national to sub-national scales to characterize the diverse and changing nature of blue food consumption. It also compares consumption patterns for fish and terrestrial meat that are potential substitutes in demand. An assessment of blue food demand across geographies and time horizons provides insight into the nutritional and environmental outcomes of changing diets, as discussed in the “Results” section.

The conceptual framework for this study aligns with consumer theory^6,7^ characterizing blue food demand as a function of population, income, relative prices, and preferences; other household characteristics such as employment and urban versus rural residence are embedded in preferences. Consumption is determined by a two-step budgeting process wherein consumers first allocate expenditures among separate groups of goods (for example, food versus non-food) and then allocate spending within each group (for example, different types of fish or fish versus terrestrial meat). Food typically comprises a large budget share for low-income consumers, making their food purchases more responsive to changes in prices and income than wealthy consumers. Accordingly, the income elasticity of demand for food in the aggregate, a metric of the responsiveness of demand to changes in income (see “Methods”), is higher for low-income populations than for high-income populations and declines with income growth (Engel’s Law)^8,9^.

Consumers diversify food expenditures according to price and quality as their incomes increase, spending less of their budget on staple foods and more on luxury items^10^. Income elasticities of demand are thus greater for high market-valued foods, including aquatic and terrestrial animal products, than for low market-valued staple foods^9–11^. Since some wild fish are used for fishmeal and fish oil in animal feeds, demand for fish as a feed ingredient is expected to rise with per capita income growth^5^. These relationships provide a foundation for assessing both time series and cross-sectional trends in blue food demand within the global food system.

The availability and affordability of blue foods also influence demand^12^. Small island nations with an abundance of wild fish in their ocean territories record especially high per capita fish consumption (Supplementary Table 1). In other regions, particularly throughout Asia, the expansion of aquaculture has driven down real prices for farmed fish produced in large volumes, making them increasingly accessible to low-income consumers^13^. Meanwhile, wild capture fish have become more expensive, both in real terms and relative to farmed fish, often restricting their accessibility to wealthier consumers^14–16^. Our projections of future demand assume that producers are able to supply the quantity of fish demanded at constant real prices (see “Methods”), a plausible assumption given the steady growth in global aquaculture production^17–19^. Climate change raises significant uncertainties surrounding this assumption, however, as described in the Discussion section.

Given the geographic patchiness of wild fish and aquaculture production, trade is critical for meeting fish demand in many parts of the world. Fish imports are especially important in countries where per capita fish demand is rising, aquaculture is limited, and wild fish capture for domestic consumption is stagnant or declining^20^. Seafood is among the most highly traded commodities in the global food system^21,22^ and has become increasingly globalized, with trade approximately doubling in terms of quantity and value from 1998 to 2018^23^.

The interaction of income, prices, and preferences drives dietary diversification and substitution in demand for different animal source foods, including fish, across countries^11^. Preferences are shaped by geographic location, dietary history, culture, time constraints, out-of-home consumption, nutritional knowledge, health and sustainability concerns, and other social and behavioral dynamics^7,24–27^. Geographies with long coastlines or significant inland water systems have strong traditions of blue food consumption^24,28^. As the food share in total expenditures declines with income growth, the role of preferences in consumer choice rises^7,25^. Fish products are now consumed in non-traditional ways, such as sushi and sashimi^29^, and markets for sustainably rated or certified fish are expanding^18,30^, reflecting income growth and changing tastes. Diverse preferences have fostered use and trade in by-products, not just edible fish or fish fillets^31^. For example, salmon heads from Norwegian and Scottish aquaculture find viable markets as human food throughout Southeast Asia, and shrimp shells and pangasius oil from Vietnam are used in medical and agricultural products (chitosan) and livestock feed formulation, both domestically and after the edible fish products are exported to China^31,32^. Fish processing wastes are increasingly used to produce fishmeal for aquaculture and livestock feeds^18^. Although our analysis focuses on patterns of fish consumption for human foods, the overall demand for aquatic animals, algae, and plants also encompasses a wide array of industrial uses and animal feed products^31^.

This study covers three tiers of analysis: global, as represented by 72 countries drawn from all continents and constituting over 80% of all blue food consumption; regional, based on the two largest fish consuming countries in each of five continents -- Asia, Africa, South America, North America, and Europe—accounting for 55% of global consumption (each continent comprising 5% or more of the global total); and national, through four country-level investigations (China, India, Nigeria, and Chile) that highlight the roles of income, trade, geography, culture, and preferences in blue food demand. Consumption is captured by apparent consumption data taken from FAO food balance sheets^33^ and does not directly measure dietary intake. Edible conversion weights and income elasticities from the literature are used to estimate current and future blue food and to compare fish to terrestrial meat consumption (see “Methods”). The terms “blue foods”, “seafood”, and “fish” are used interchangeably throughout this study to denote marine and freshwater finfish (including diadromous fish), crustacean, and mollusc species, as reported by FAO (Supplementary Table 2). Although aquatic plants, seaweed, and aquatic animals other than fish and shellfish are important for food and nutritional security in certain locations, they are not included in our analysis due to data limitations.

In this work, we show that global demand for blue foods has roughly doubled since the turn of the 21st Century and will likely double again by 2050 assuming constant real prices for fish. Understanding which blue foods people eat and where these products are consumed is more complicated, however, and requires a deeper understanding of the wide diversity of fish produced and traded around the world, and the types of blue food products consumed across geographies and by different income groups. Our study concludes that the nutritional and environmental impacts of rising blue food demand will depend on substitution among fish groups and other animal source foods in national diets.

## Results

### Global fish consumption

Our global analysis focuses on population and income per capita as major determinants of blue food consumption and assumes that supply is not constrained. During the 20-year period from 1998 to 2018, global average fish consumption per person rose from 15.6 to 20.4 kg/year on a live-weight basis, and from 11.5 to 15.1 kg/year in edible weight^33^. Multiplying these figures by the growing population, the aggregate volume of fish demand (live-weight) increased from 93.6 to 152 million tonnes (Mt).

Income growth and associated changes in dietary habits have been shown to be a more influential driver of fish demand in recent decades than population growth at the global scale^3,5^. Cai and Leung^3^ disaggregated the effects of population growth and consumption per capita for the period 2008–2013 and found that they accounted for 40% and 60%, respectively, of the increase in global fish demand. Their results showed considerable regional variation: in Sub-Saharan Africa the contribution of population growth to total demand was 90%, while in East Asia (mainly China) where economic growth was much stronger and population growth relatively weak, population accounted for only 13% of the overall increase in fish demand. Asia was the source of virtually all of the increase in global fish consumption over this period, suggesting that Asian countries are undergoing a significant dietary transition, one that is just beginning in Sub-Saharan Africa.

**Fig. 1:**
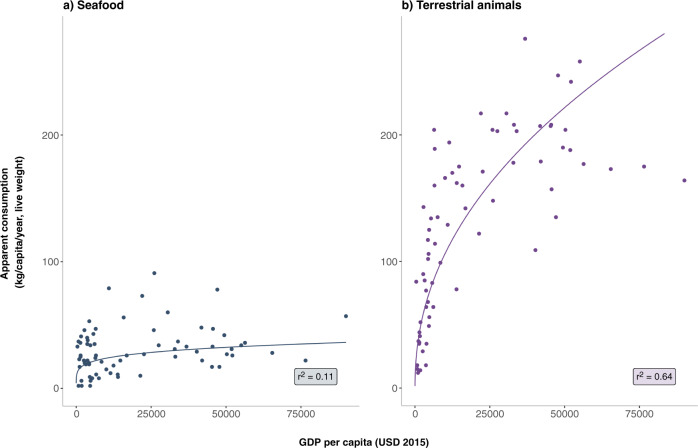
Role of income in global seafood and meat consumption. Relationship between apparent consumption (live weight) and GDP per capita for seafood (dark blue dots) and terrestrial animals (purple dots). Line of best fit modeled as y = ax^b^. **a** Per capita fish consumption (y_fish_) as a function of per capita GDP (x) (y_fish_ = 4.11x^0.19^; *r*^2^ = 0.11; *r* = 0.33). **b** Per capita terrestrial animal consumption (y_animal_) as a function of GDP per capita (x) (y_animal_ = 1.56x^0.46^; *r*^2^ = 0.64; *r* = 0.80). The relationship between per capita consumption and GDP per capita is significantly weaker for fish than for terrestrial meat. Countries with very high per capita consumption of fish (>50 kg/cap/year, live weight) include French Polynesia, Maldives, Fiji, Antigua & Barbuda, Iceland, Malaysia, Barbados, Lithuania, Spain, South Korea, Portugal, and Norway. Data represent 2015 values for 72 countries with GDP and population data from The World Bank (https://data.worldbank.org/) and apparent consumption from FAOSTAT^23^.

To assess the role of income in blue food consumption globally, we use an aggregate metric for fish following the demand model of Muhammad et al.^2^ and compare per capita apparent consumption in relation to income for fish vs. terrestrial meat with cross-sectional data (see “Methods”). Our analysis reveals a weak relationship between per capita fish consumption and income across the 72 countries in our global dataset (*r*^2^ = 0.11) (Fig. 1). The best-fit model in Fig. 1 shows a significantly stronger relationship between consumption of terrestrial meat and income (*r*^2^ = 0.64), reflecting the nutrition transition whereby meat consumption rises steadily with income growth and tapers off at higher levels of income^11^. These results are consistent with estimates by Muhammad et al.^2,34^ showing a uniformly higher income elasticity of demand for meat than for fish.

A key explanation for the weak relationship between income and fish consumption per capita is that “fish” comprise thousands of different species captured or cultivated in a wide array of freshwater and marine systems. The diversity of seafood consumed globally is substantially higher than that of terrestrial meat, which is dominated by a relatively small number of cattle, poultry, and swine breeds. Previous models of fish demand have shown that consumption of blue foods cannot be characterized by a single ‘fish’ variable, as preferences and income elasticities of demand differ widely between low versus high market-valued fish^1,3,5^. Fish consumption also tends to be measured less accurately than terrestrial meat in FAO data due to the wide variety of edible products^35^. Preserved blue foods (dried, salted, fermented, smoked) account for a substantial share of fish consumption, particularly in Southeast Asia and Africa, adding nutrients and distinct flavors to local cuisines^20,36–38^. In the Mekong River Basin, small quantities of dried or otherwise processed fish account for 15% of fresh whole animal equivalent weights^39^, and in Myanmar they constitute roughly one-third of all blue food consumption^14^.

**Fig. 2:**
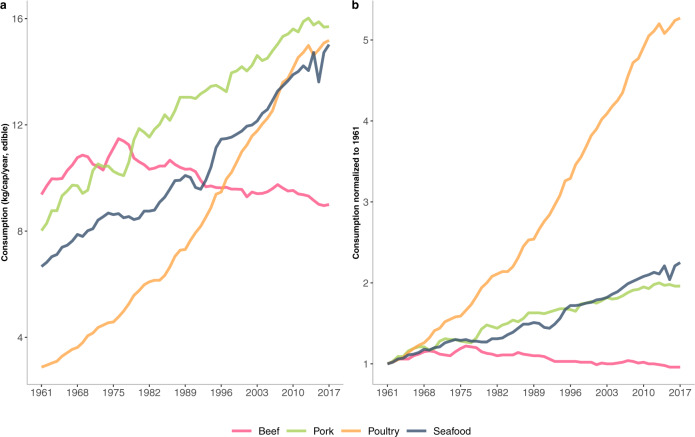
Global meat and fish consumption in edible weight from 1961 to 2017. **a** Per capita animal consumption by type. Annual per capita consumption of poultry increased five-fold from ~3 kg to 15 kg, while that of seafood more than doubled from ~7 kg to nearly 16 kg. Global annual per capita consumption of beef has declined since the mid-1970s. In 2017, in absolute terms, per capita seafood and poultry meat consumption converged and pork consumption leveled off. **b** Normalized global per capita animal consumption by type from 1961 to 2017, pegged to 1961. Annual growth rates of poultry meat, seafood, pork, and beef during this period were 3.4%, 1.6%, 1.4%, and −0.08% respectively. Data Source: FAOSTAT^23^.

Terrestrial meat and fish are often grouped together in analyses of diet diversification^9,11,35^, yet disaggregation by beef, pork, poultry, and seafood is critical for understanding substitution in demand. During the past 60 years, poultry consumption has increased while beef consumption has declined; between 1961 and 2017, global annual growth in edible per capita consumption was 3.4% for poultry, 1.6% for seafood, 1.4% for pork, and −0.8% for beef (Fig. 2). Although substitution of fish for beef has been advocated on health and environmental grounds^40,41^, poultry appears to have already served as a major substitute for beef in global diets. Certain fish species, such as salmon and shrimp, are similar to poultry in industrial organization, processing techniques, and nutritional and cuisine attributes^42^. Global per capita fish and poultry consumption has converged at 15–16 kg/cap/year in edible weight (Fig. 2a). Prices for the two commodities are roughly equivalent (as measured against the price of staple calories) in certain large countries undergoing nutritional transitions, such as China and India, but show greater divergence in other countries that have varied availability and tastes for fish versus poultry^43^. Preferences have long been recognized as a major factor in consumer choice of animal source foods^7,44^, highlighting the need for further analysis at regional to sub-national scales where geography, culture, and tastes can be differentiated.

### Regional fish consumption and trade

**Fig. 3:**
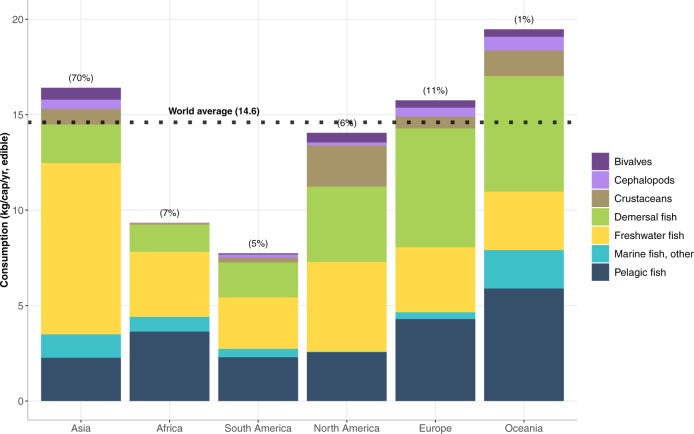
Per capita apparent fish consumption by region and species group (kg/cap/year, edible weight) in 2015. Numbers in parentheses above the bars represent the share of global consumption. Asia, Europe, and Oceania demonstrate a high level of edible fish consumption per capita in relation to the global average (14.6 kg/cap/year, edible). Fish consumption in North America is close to the global average, whereas Africa and South America are significantly below the global per capita average. Data Source: FAOSTAT^23^.

To capture the regional heterogeneity of blue food demand, we disaggregate fish consumption into seven major species groups: freshwater fish (including diadromous fish such as salmon), demersal (e.g., cod, halibut), pelagic (e.g., tuna, forage fish), other marine fish, crustaceans, cephalopods (e.g., squid, octopus), and bivalves (Supplementary Table 2). There are distinct patterns of seafood consumption across regions (Fig. 3): for example, relatively large shares of freshwater fish consumption in Asia, pelagic and freshwater species consumption in Africa and South America, and demersal species in Europe, North America, and Oceania. Per capita fish consumption in Asia, Europe, and Oceania exceeds the global average, whereas Africa and South America are well below the global average.

**Fig. 4:**
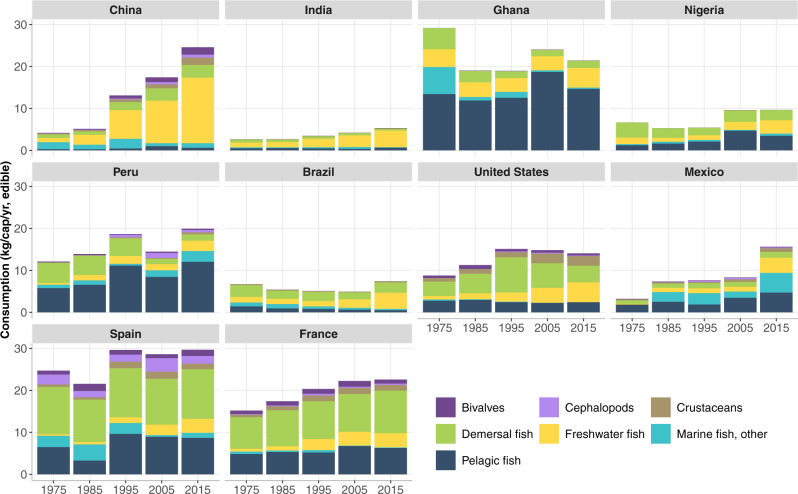
Per capita fish consumption by region for selected species groups (kg/cap/year, edible weight) from 1975 to 2015. Asia (China and India)**:** Widespread consumption of freshwater fish, but China is also a large global consumer of bivalves, crustaceans, and demersal fish. Africa (Ghana and Nigeria): High consumption of small pelagic fish and freshwater species. South America (Peru and Brazil): All categories of fish species consumed, with high consumption of pelagic fish in Peru. Europe (Spain and France): Wide mix of fish groups consumed, with strong preference for demersal fish, followed by pelagic and freshwater fish and bivalves. North America (USA and Mexico): All categories of fish species consumed with high consumption of crustaceans in the USA relative to most other countries in the regional analysis. Each continent represented in the figure accounts for 5% or more of global consumption as indicated in Fig. 3 and thus exclude Oceania, which accounts for only 1% of global consumption Data Source: FAOSTAT^23^.

Viewing consumption patterns over time for individual countries within regions (Fig. 4) provides additional insight into the roles of preferences, availability, and economic determinants of fish demand. This set of countries is confined to continents accounting for 5% or more of global consumption and includes those with high per capita consumption—up to 30 kg/year, twice the global average—and those with relatively low per capita consumption but large populations. A cursory look at the types of fish consumed in each region demonstrates that China is the dominant consumer of freshwater fish, while Ghana and Peru are large consumers of pelagic fish (especially small forage fish). Fish consumption in USA, Mexico, Spain, and France is highly varied, with relatively large consumption of high market-valued demersal fish, crustaceans, pelagic fish (including tuna) and other marine fish. Per capita consumption of bivalves is greatest in Spain, France, and China.

**Fig. 5:**
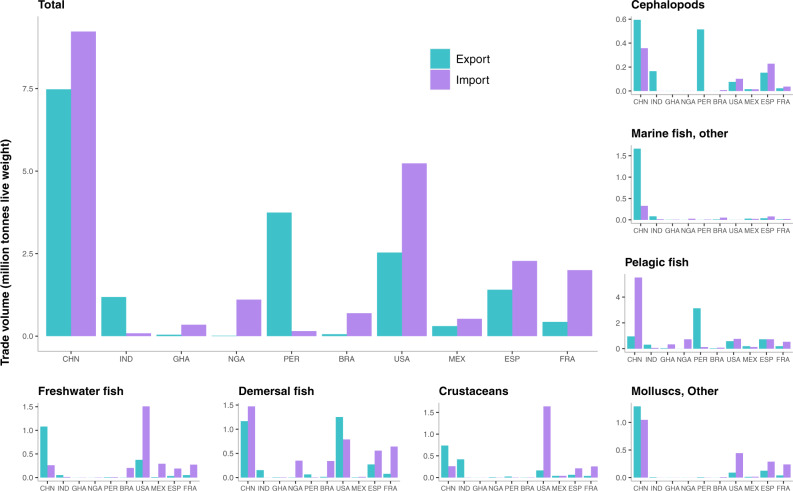
International fish trade in 2015. Data show fish and fishery product for seven species groups and their sum (in million tonnes, live weight) for 10 countries in our regional comparison set. Note that the displayed data includes fishery product trade not destined for human consumption, leading to particularly high exports of pelagic fish from Peru and imports of pelagic fish by China. Data Source: FAO Food Balance Sheets^33^.

Delineating fish by species group is also critical for understanding trends in fish trade, as demand is met by both domestic production and imports. The volume of fish trade for the seven species groups in our 10-country regional dataset reveals several interesting patterns (Fig. 5). China is the leader in total fish exports and imports; it also dominates in several specific categories of fish trade, such as mollusc, freshwater, and other marine fish exports, and demersal fish imports. USA, France, and Spain are also large importers of most species, with the exception of pelagic and other marine fish. USA, a large producer of farmed catfish during the past half century, has become the world’s leading importer of freshwater fish as well as crustaceans. Although India has the lowest per capita consumption of fish in our regional dataset, it is a net exporter of fish overall and second only to China in crustacean exports. As fish supplies increase in India, however, consumption per capita is also rising^13,45^. Demand for higher market-valued marine and freshwater species, both farmed and wild, is increasing in several Asian countries as production and incomes rise, gradually redirecting products once produced mainly for export to domestic markets^13,18,46,47^.

The dominance of certain countries in international fish trade masks the importance of trade for Africa, where imports of frozen fillets and small pelagic fish have been rising as production from domestic fish capture has either stagnated or been exported^20^. Fish demand has grown faster than supply in Africa, resulting in an increase in the import share of consumption from 16% in 1970 to 39% in 2017—a major jump given that the import share for food in aggregate was 13% in 2017^20^. In Nigeria, fish have traditionally been among the cheapest animal source foods, but limited domestic supplies and rising imports of frozen fillets have caused real fish prices to rise significantly during the past decade^20^.

Real prices of blue foods, particularly relative to terrestrial animal products that substitute in demand, are important determinants of consumption, with low-income households being more responsive to price than wealthy households^1,3,5,8^. Global price indices for fish generally represent traded commodities^12,19^ and do not fully capture the diversity in species, processing, and quality across geographies and countries at different stages of economic development (Supplementary Fig. 1). Demand models that estimate consumer prices by dividing expenditures by quantity consumed without accounting for quality produce biased results^10^. Prices have been recorded for a wide variety of standardized fish and meat products through the World Bank International Comparison Program (ICP) in 2011 and 2017^48^. During this period, the price of fish rose in all 10 countries in our regional comparison set, while meat prices were more stable overall, particularly relative to fish (Supplementary Table 3). Using ICP data to calculate the relative caloric price of seafood to staple grains, Headey and Alderman^43^ found that fish are comparatively expensive in low-income countries and cheap in high-income countries. In Asian countries where availability is high, fish are an affordable source of animal source foods^43^.

### Country-specific examples

**Fig. 6:**
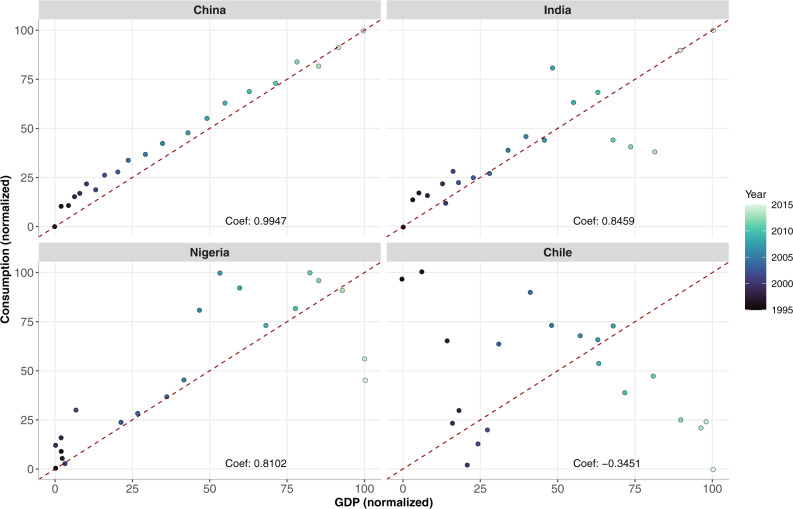
Per capita fish consumption relative to GDP per capita for four case study countries from 1995 to 2015. The case study countries include China, India, Nigeria, and Chile. Both GDP per capita and per capita apparent fish consumption were normalized between 0 and 100 for comparability across countries. Each year is represented by a blue dot. China shows a strong positive correlation between consumption per capita and GDP per capita (*r* = 0.99); India and Nigeria show a moderately high positive correlation between fish consumption per capita and GDP per capita (*r* = 0.85 and 0.81, respectively), with greater variation at mid to high income levels; Chile shows a negative correlation between consumption per capita and GDP per capita (*r* = −0.35), with fish consumption falling as income rises. Data Source: FAOSTAT^23^.

Case studies of China, India, Nigeria, and Chile provide further insight into geographic patterns of blue food demand at national and sub-national scales. China and India were selected on the basis of their large populations, strong growth in GDP per capita in recent decades, and sizeable roles in global fish production, consumption, and trade. Nigeria has relatively low per capita fish consumption but contains the largest population in Africa, projected to exceed 400 million by 2050, surpassing USA as the world’s third most populous nation^49^. Although Chile is much smaller than the above-mentioned countries, it serves as an interesting case given its export orientation and substitution of terrestrial meat for fish in national diets. Across these four countries, the relationship between income growth and fish consumption per capita is varied (Fig. 6).

China is the largest producer, consumer, processor, and exporter of fish globally, and its imports of fish have been rising in recent decades^46,47,50^. Per capita consumption of fish (edible weight) increased five-fold between 1975 and 2015 (Fig. 4) and is projected to rise by almost 50% from 2015 to 2050 (Fig. 8). More than 95% of China’s fish production and domestic consumption is concentrated in its eastern, southern, and central provinces^51^. Chinese National Statistics reported per capita fish consumption of 16.7 kg/yr and 9.6 kg/cap for urban and rural areas, respectively, in 2019, with fish consumption exceeding 25 kg/cap/year in eastern cities and as low as 1 kg/cap in western provinces^51^. It is worth noting that urban fish consumption may be underestimated by 25–30% because government reports omit out-of-home consumption^52,53^.

**Fig. 7:**
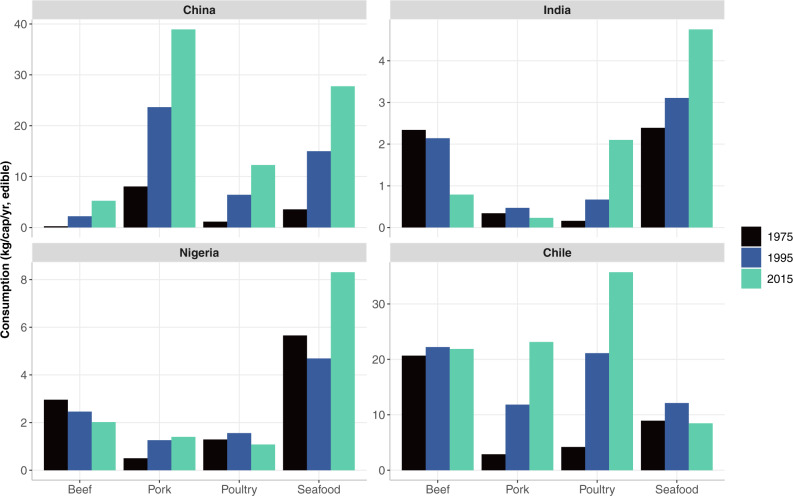
Per capita meat and seafood consumption for four case study countries in 1975, 1995, and 2015. Meat is disaggregated by beef, poultry, and pork. Seafood includes all freshwater and marine species of finfish, crustaceans, cephalopods, and bivalves in edible weight with conversion from live-to-edible based on conversion factors reported in the “Methods” section. Note the differences in scales along the y-axes, indicating that India and Nigeria consume significantly less seafood and meat on a per capita basis than China and Chile. Seafood consumption per capita has risen in all locations except for Chile, where terrestrial meat consumption (particularly poultry and pork) has increased significantly. China has higher consumption per capita of pork than seafood. Data source: FAOSTAT^23^.

Freshwater aquaculture systems have supported local demand for fish in China for centuries and have expanded under government incentive programs since the mid-1980s when the country’s capture fisheries became over-exploited. With its long coastline, local marine fish such as yellow croaker have long been part of Chinese diets, but consumption of freshwater fish, molluscs, and even non-traditional salmon products has risen with domestic aquaculture expansion^18,54^. Output from aquaculture (both freshwater and mariculture) exceeded that of wild fisheries in 1988 and now contributes over three-quarters of China’s total fish production^50^. Consumption of marine species has tripled during the past 30 years, and the gap between inland and coastal consumption of marine fish has narrowed with advances in supply chain logistics and cold chain technology^55^. Consumption of all animal source foods has increased in China since 1975 (Fig. 7). Although pork remains the dominant animal source food in the average Chinese diet, the demand for fish has been rising with income growth and is often preferred as a healthy food to terrestrial meat^56^. Demand for eco-labeled seafood in China is also emerging^57^.

India is the second-largest producer and consumer of fish globally and a net exporter, although consumption per capita is well below the world average (Supplementary Table 4). National Sample Survey Reports from 1987–88 to 2011–12 show that average consumption increased from 1.92 to 2.64 kg/cap/year (live weight) and the share of households consuming fish regularly rose from 27 to 32%^58,59^. Two-thirds of all households in India, however, reported eating no fish at all in 2011–12^59^. Annual per capita fish consumption estimated from national survey data was below that published by FAO (Fig. 4), underscoring major estimation and reporting inconsistencies at the national scale.

National Sample Surveys reveal substantial variation in fish consumption among states in India^59^. Ninety percent of all households in Assam, Kerala, and West Bengal eat fish regularly, compared to less than 2% of households in Himachal Pradesh, Haryana, Punjab, and Rajasthan. In states where fish is not a common food, urban and wealthier households report lower consumption than their rural and poorer counterparts. Fish consumption is also higher in Muslim versus Hindu households, and in Schedule Tribes versus upper caste households. Households that do not consume fish typically adhere to vegetarian diets and are significantly less likely to report consumption of meat (beef, chicken, mutton, or pork) or eggs. Fish consumption is more common in the predominantly rice-eating southern and eastern states—mainly due to agroecological conditions, availability, and regional cuisines—than in the northern and western parts of the country where wheat is the staple food^24^.

Given wide inter-state variation, national-scale projections may misestimate future fish consumption in India. Existing projections use single values for income and price elasticities of fish demand for the entire country^1,5,45^ despite large differences in consumer behavior among states (e.g., authors’ calculations from^59^ indicate the income elasticity of demand for fish is substantially higher in eastern and southern states than in northern states). For India as a whole, poultry consumption has increased in recent decades along with fish, whereas beef and pork consumption have fallen (Fig. 7). Milk consumption per capita (not shown in Fig. 7) was significantly lower for households where fish was consumed across all income classes in 2010–11^59^. In addition, butter, ghee, and eggs have been shown to be substitutes for fish in Indian diets^60^.

In Nigeria, edible fish consumption was estimated at 10 kg/cap/year in 2015 (Supplementary Fig. 5), slightly higher than the regional average for Africa of 9 kg/cap/year and below the global average of 14.6 kg/cap/year^33^. Fish plays a major role in the average Nigerian diet with roughly half of the population eating fish two to three times per week^61^. Like India and China, however, there are sub-national differences, with households in southern Nigeria consuming about twice as much fish as those in the north^20^. Per capita fish consumption is higher in urban than rural households, particularly for fresh (versus dried) fish, and middle- and high-income households eat fish more frequently than do lower-income households^62^. Households in the south also consume more frozen imported fish than in the north. Higher levels of fish consumption in the south reflect its proximity to the coast, and hence historical preferences and access to imports, as well as the region’s relative success in aquaculture development^20^.

Per capita consumption of fish exceeds terrestrial meat in Nigeria, although pork consumption has risen in recent decades (Fig. 7), mainly in the non-Muslim southern region. Local studies suggest that affordable, small pelagic, and preserved fish (dried and smoked) are most widely consumed, but imported frozen fish now comprise the largest budget share of seafood consumed across the country on average^20^. Given steady growth in fish demand, Nigeria has invested heavily in aquaculture production (mainly freshwater catfish), accounting for 52% of Sub-Saharan Africa’s farmed fish output^63^. Replacing locally diverse indigenous species with farmed freshwater fish has mixed nutritional consequences^37^, but having access to affordable fish in the diet is critical for Nigeria’s nutrition security overall^20,36,43,64^.

In Chile, fish consumption has traditionally played an essential role in national diets given the country’s a long coastline and extensive freshwater bodies in the south. An estimated 55% of high-income and 38% of low-income households consume fish weekly^65^. Despite Chile’s culinary history, per capita consumption of fish has fallen during the past two decades while consumption of poultry and pork has increased (Fig. 7). A shift in dietary preferences and a re-orientation of domestic capture fisheries toward global markets help to explain this trend.

Chile is the second largest producer and leading exporter of mussels globally^65^. Expansion in the mussel industry has been driven by strong international demand, mainly by European countries, which together with Russia and the U.S., accounted for almost 70% of Chilean mussel export volume in 2019^66^. Domestic fish consumption has decreased in response to rising prices over the past 15 years, a trend that artisanal fishing communities are trying to reverse^67^. Local consumption of certain mollusc species sold in luxury export markets, such as the gastropod “*Loco”* (*Concholepas concholepas*), has fallen dramatically^68^. Salmon aquaculture in Chile is also export-orientated, with output destined mainly for markets in high-income nations such as the U.S., Japan, and EU^69^. Chilean consumers have not yet demonstrated a strong preference for farmed salmon despite large-scale domestic production^70^.

National health and environmental consequences of shifting diets from fish to terrestrial meats^60^, in addition to the environmental implications of expanding intensive salmon aquaculture to meet rising international demand^71^, illustrate the influence of global markets on nutrition and sustainability outcomes in countries like Chile that are significantly engaged in blue food trade.

### Fish consumption to 2050

To assess future fish demand, we calculate growth in fish consumption, disaggregated by species group, for the 10 countries in our regional dataset from 2015 to 2050 based on projections of population and income growth (see “Methods”; Supplementary Table 4). Real prices are held constant as we do not create a closed model that solves for price endogenously with supply estimations and assume instead that production keeps pace with demand. Our projections do not represent a linear extension of 2015 consumption patterns, as consumption of different types of fish will change with income growth depending on the relevant income elasticity of demand, typically favoring higher market-valued products as countries become richer^11^.

**Fig. 8:**
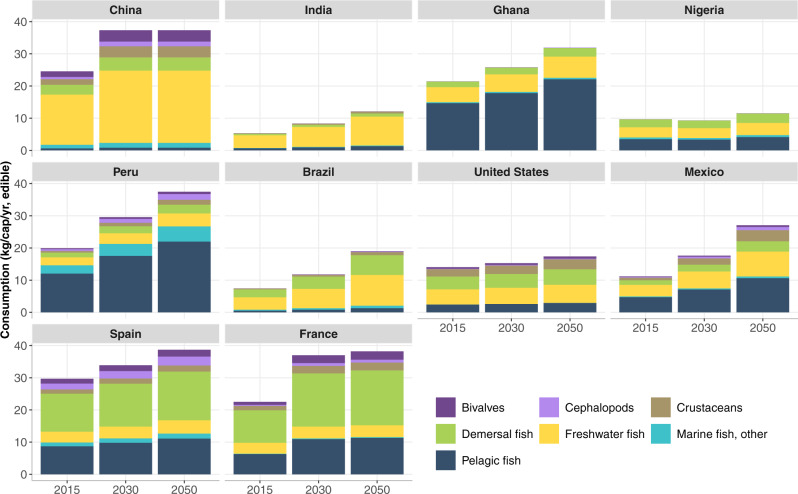
Per capita fish consumption by species groups in the 10 countries in our regional comparison set (kg/year, edible weight), 2015–2050. Future consumption is based on projected growth in population, income per capita, and income elasticities of demand by species group (see “Methods”; Supplementary Table 4). Asia: Freshwater fish and overall consumption continue to grow across all countries, but per capita consumption remains relatively low in India. Africa: Pelagic fish continue to play an important role in fish-consuming countries. Nigeria’s slow recovery from an economic recession in 2016 and COVID dampen predicted consumption. South America: Pelagic fish consumption dominates in Peru, whereas Brazil mainly consumes freshwater and demersal species with lower consumption per capita. North America: Per capita fish consumption is varied by species and is predicted to remain at moderate levels in the USA and show more significant growth in Mexico. Europe: Fish consumption in Spain and France is highly varied and remains relatively stable at the current high consumption rate. Data on fish consumption per capita in 2015 are taken from FAO Food Balance Sheets^33^.

**Fig. 9:**
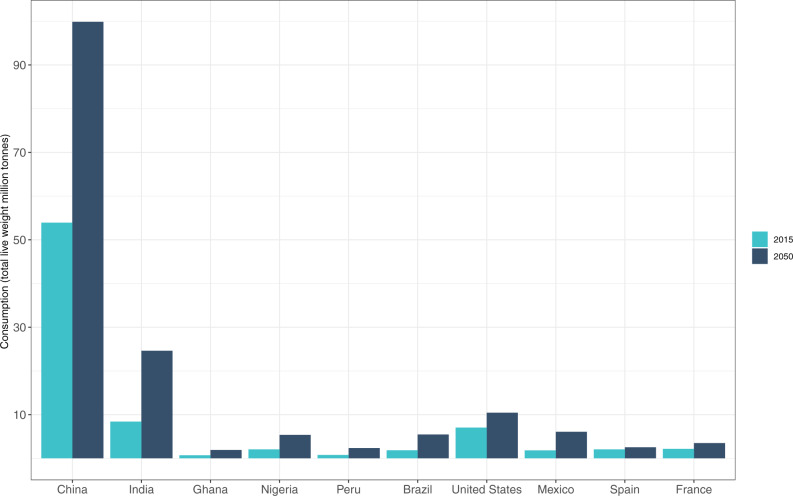
Total fish demand for human consumption (million tonnes, live weight) for the 10 countries in our regional dataset in 2015 (light blue) and 2050 (dark blue). 2050 projections are based on estimates for population and income growth and income elasticities of demand for the 7 species groups in our regional analysis (see “Methods”; Supplementary Table 4). China remains the largest fish consumer and is expected to account for over half of the growth in total fish consumption within this set by 2050. Total live-weight fish demand is also projected to double in India, Nigeria, Brazil, and Mexico by 2050. Data source: FAO Food Balance Sheets (2020)^33^.

Our projections indicate that per capita fish consumption, measured in edible weight, will increase in all areas, more than doubling from the current global average consumption of 15 kg/cap/year^33^ in half of the 10 countries by 2050 (Fig. 8). Asia will continue to lead in freshwater fish consumption and will have the highest demand for fish overall in 2050 (Fig. 9). China will remain the world’s largest fish consumer, vastly surpassing all other countries in the regional dataset. Total fish demand (live weight) in India, the second largest consumer, is projected to increase from 6.2 Mt to 19.8 Mt due to its large and growing population (~1.6 billion by 2050^49^). Projections based on FAO data may overstate India’s future fish consumption as discussed earlier, but even at half the projected national per capita consumption level, India’s population-driven demand will be large. Projections show China consuming a diverse set of species in 2050 including crustaceans, demersal fish, and cephalopods, while Ghana and Peru will continue to dominate the consumption of small pelagic fish (Fig. 8). Europe, North America, and South America will continue to consume a wide variety of species. Per capita edible fish consumption in Ghana is expected to be roughly triple that of Nigeria in 2050, but given the size of Nigeria’s population, its aggregate edible fish consumption is projected at 4.6 Mt in 2050, significantly higher than Ghana’s at 1.7 Mt (Supplementary Table 4).

Overall, our projections indicate that aggregate edible consumption for the sum of countries in our regional model will increase by over 80%, from 54.7 Mt to ~100 Mt, between 2015 and 2050 (Supplementary Table 4). These countries comprise 55% of global fish consumption today and are thus indicative of the degree of change in future fish consumption worldwide. On a live-weight basis, total demand for fish across the 10 countries will almost double from 80.7 Mt to 154.6 Mt. The model provides a rough gauge of future fish demand but is only suggestive as it does not include changes in dietary preferences for different types of fish and other animal source foods. Preferences will likely change for a variety of reasons such as consumer concerns over human health, sustainability, food safety, convenience, and the emergence of plant-based meat substitutes.

### Nutritional and environmental consequences of demand

Looking forward to 2050, the nutritional and environmental consequences of fish demand will depend largely on the quantity and types of fish species consumed and substitutions in demand between fish and other animal source foods. The dietary transitions unfolding globally will serve to restructure food systems and will likely set certain countries onto particular nutritional trajectories^72^. Although projections in Fig. 8 rely on a partial structural model of demand, they suggest a significant rise in fish consumption from current levels. As blue food consumption increases in several of these 10 countries (i.e., China, India, Nigeria, USA, Peru, Mexico, Brazil), per capita consumption of meat, dairy, and eggs will likely decrease, leading to greater dietary intake of DHA + EPA fatty acids, iron, calcium, and vitamin B-12, but reduced vitamin A intake if aquatic foods are substituted for other meat and dairy products^60^. If preferences were also to shift toward aquatic foods and away from ultra-processed foods at higher levels of income for health reasons, consumers would likely experience reduced rates of diet-related non-communicable disease (e.g., cardiovascular disease, diabetes, stroke)^60^ provided that they have access to affordable fish supplies. For most countries in Fig. 8, rising fish consumption will enrich diets in terms of micronutrients, including iron, calcium, vitamin B12, and zinc, and seafood omega-3 fatty acids^60^.

Dietary transitions will also influence environmental outcomes at local to global scales. The EAT-Lancet Commission report in 2019 concluded that sustainable and healthy diets for the global population require a shift away from beef and foods high in saturated fats and sugars, and towards nutritious foods such as fish, vegetables, nuts, and fruits^40^. Substituting seafood for terrestrial meat for a growing world population will be accomplished mainly through aquaculture expansion, although capture fisheries, if managed sustainably, also have the potential to add to future fish supplies^41,73^. Aquaculture production is more geographically concentrated than capture fisheries, and thus an increase in the share of aquaculture in the total fish supplies will require more seafood trade or further development of aquaculture into new geographic areas^17^.

Aquaculture has become progressively more sustainable over the past 20 years, yet environmental challenges persist^18^. As aquaculture intensifies in both freshwater and marine systems, feed formulations will depend increasingly on terrestrial crop production and will continue to rely on marine resources^18,71^. Bivalves are widely advocated as a sustainable seafood option, but it is unclear how dietary preferences will evolve for this group of species. Global demand for sustainable seafood will help shape future consumption patterns, yet such demand has been dominated to date by North America, Japan, and Europe and does not capture the breadth of global seafood demand described here^74^. Although the quantity of certified seafood has grown substantially in recent decades, future expansion is uncertain given the lack of inclusion of small-scale producers in many areas including Asia^30^.

The degree to which aquatic food systems will be able to keep up with changes in demand also depends on their ability to maintain production in a rapidly changing climate^75–77^. The prospects of climate-driven constraints on blue food supplies mean that seafood prices could rise and become more variable in the future, impacting fish consumers, especially the poor who spend a disproportionate share of their income on food^77,78^. Low-income consumers typically respond to rising prices and declining purchasing power by decreasing overall food consumption, shifting from nutrient-rich to energy-rich foods, and substituting from high- to low-quality starchy staples^79^. Intersecting issues related to environmental change, food equity and justice, and nutrition will thus be important to address in the coming decades.

## Discussion

This paper highlights the need to go beyond the use of a single category to represent all blue foods in analyses of global food demand. It also questions persistent assumptions about the relationship between income and fish consumption and underscores the importance of analyzing socio-cultural drivers of demand across multiple species and geographic scales. The paper demonstrates the geographically diverse and highly dynamic nature of blue food consumption and identifies four major knowledge gaps that require attention for future analyses of blue food demand.

First, although national-level data on blue food production and trade are widely available, data and information are lacking on consumption as measured by dietary intake. Opportunities exist for harmonizing and improving data collection at sub-national to global scales, and for resolving differences between national household survey data on reported consumption and FAO food balance sheet data on apparent consumption.

Second, urbanization appears to be an important driver of blue food demand, but existing studies show mixed results on this relationship when controlling for income growth across countries. A comprehensive empirical analysis of the role of urbanization in blue food demand is needed to identify structural features and dietary preferences underlying this relationship, including the role of out-of-home consumption.

Third, our analysis omits aquatic plants, seaweed, and other aquatic animals (e.g., amphibians, reptiles) due to insufficient data at national scales. Data on direct human consumption of seaweed versus industrial use is incomplete, and aquatic plant production is recorded by FAO under the ill-defined category, “aquatic plants nei (not elsewhere included)”^18^. This paper calls for a more inclusive treatment of blue foods than previous demand models given the significant role of aquatic foods other than fish in food and nutrition security in many locations.

Finally, our analysis does not explicitly include price dynamics as derived from supply-demand balances, given that the study focuses specifically on blue food demand. Price and affordability are the main barriers to nutritious diets for poor consumers^64^. The FAO has created a global fish price index based on traded commodities which serves as a proxy for both traded and non-traded fish prices^19,80^; however, this index and others do not target the wide range of locally produced and consumed species that are important for nutrition security throughout the Global South (Supplementary Fig. 1). National data on fish prices should be improved and coordinated for policy purposes given the nutritional and food justice consequences of price hikes for food-insecure populations.

More generally, there is an urgent need to integrate blue foods into national and global strategies on nutritional security and sustainable food systems. The dominant shock to blue food systems during 2020-21 has been the global COVID-19 pandemic, a topic not covered in this paper. Much improved data on household consumption and prices of both raw and processed products (e.g., dried or salted for long shelf life) are urgently needed to ameliorate the impacts of shocks, including pandemics and climate change, on vulnerable populations. Only then can global food policy leaders begin to create an internationally coherent vision of blue foods -- one that has policy relevance and can deliver on nutrition, equity, and environmental objectives.

## Methods

### Historical consumption: global aquatic and terrestrial animal source foods

Historical consumption data are drawn from the “Food” category in the FAO Food Balance Sheets^33^. Data on beef, poultry, and pork are in dressed carcass weight (ready-to-cook weight). Apparent fish consumption for all species is reported in live weight equivalent in the Food Balance Sheets. To analyze the relationship between apparent aquatic and terrestrial animal source food consumption and incomes, we use World Bank data on per capita GDP (at constant 2017 international $)^81^ in 2015 across 72 countries comprising over 80% of global fish consumption. We use a best fit line, modeled as y = ax^b^ to reflect the nutrition transition, and compare the per capita consumption-income relationships between fish and terrestrial animals with *r*^2^ values (*r*^2^ = 0.11 and 0.64 in the case of fish and terrestrial animal consumption, respectively) (Fig. 1).

When comparing the global per capita consumption of aquatic and terrestrial animal source foods from 1961 to 2017, we correct for live-weight quantities of fish, which overstate consumption of fish relative to other animal source foods in FAO statistics^82^. Conversions from live weight to edible weight for the FAO species groups are primarily from Edwards et al.^82^, and from FAO^83^ for cephalopods. For conversions of the aggregate “Fish, Seafood”, an average of the “Fish and Seafood” aggregate conversion rates in Edwards et al.^82^ is weighted by the contribution of aquaculture to global consumption according to the FAO (2020)^28^. Conversion rates used in our analysis are: Fish, Seafood - aggregate (0.74), Freshwater Fish (0.87), Demersal Fish (0.87), Pelagic Fish (0.87), Marine Fish, Other (0.87), Crustaceans (0.37), Cephalopods (0.70), Bivalves (molluscs, other) (0.17).

### Historical consumption of aquatic foods by region and selected countries

Our regional analyses of fish demand and trade focus on five continents that constitute 99% of global live-weight fish consumption based on FAO Food Balance Sheet data^33^: Asia, Africa, South America, North America, and Europe. (Oceania is omitted from the detailed regional analysis given that it comprises only 1% of global fish consumption.) We explore historical per capita consumption for seven fish species groups, measured in edible weight, for the top two fish consuming countries in each region (calculated by per capita consumption times population size): China, India, Ghana, Nigeria, Peru, Brazil, USA, Mexico, Spain, and France. International trade of all fish is disaggregated by the seven specific fish species groups used in direct human consumption (Mt, live weight equivalent), for these ten countries in 2015. The fish trade data were drawn from FAO Food Balance Sheets^33^ and were assessed based on the volume of individual countries’ fish exports and imports, excluding re-export and re-import data.

To analyze the relationship between per capita income growth and fish consumption within the four selected case studies (China, India, Nigeria, and Chile), historical GDP per capita from the World Bank are normalized and compared to normalized per capita fish consumption (live weight) from the FAO from 1995 to 2015. Normalized data are used for comparability given significant differences in GDP per capita across the four countries.

### Projected demand to 2050

Projected demand in global food market analyses is commonly drawn from demand-supply equilibrium models^5,84,85^. This paper, by contrast, focuses on the demand side only and uses current species-group consumption^33^, population growth^86^, predicted GDP PPP per capita growth inclusive of recent estimates of COVID-19 impacts^87–89^, and income elasticities^2,3^. Our model does not account for projected changes in income distribution^90^. We assume a perfectly elastic supply of fish; that is, producers are able to meet demand at a constant price in any given period. The model does not include own- and cross-price elasticities and thus focuses specifically on quantity shifts in demand as opposed to both quantity and price dynamics^12^.

To highlight the composition of blue foods and predicted consumption within countries and to retain global comparisons, our model is built for the selected 10 countries in our regional comparison set. Since we are interested in exploring how non-price determinants such as income and population growths influence blue foods consumption, the model is specified as follows:

1 $${C}_{{ist}+1}={C}_{{ist}}\left(\frac{{Y}_{{it}+1}}{{Y}_{{it}}}\right)+{\varepsilon }_{{ist}}\left(\frac{{G}_{{it}+1}-{G}_{{it}}}{{G}_{{it}}}\right)$$

Where ${C}_{{ist}+1}$, consumption in country *i* for species *s* in time period *t+1*, is based on consumption for the species in the previous time period, population growth $\left(\frac{{Y}_{{it}+1}}{{Y}_{{it}}}\right)$ and income growth $\left(\frac{{G}_{{it}+1}-{G}_{{it}}}{{G}_{{it}}}\right)$ scales by the income elasticity ${\varepsilon }_{}$ specific to the species *s* in the country *i* in time period *t*. Income elasticities of demand for food reflect the responsiveness of demand to changes in income and are calculated as the percent change in the quantity demanded for good *s* divided by the percent change in income for a given time period *t* and consumer population^8^.

Rather than supply constraints and prices exerting downward pressure on demand, the income elasticities in the model to 2050 were set to converge at 0.4 by 2050 in line with the global results from Muhammad et al.^2^. We assume that all income elasticities that begin above 0.4 trend towards 0.4 in equal annual increments, and that the implicit downward pressure on demand from a lower income elasticity likely captures some of the additional inhibitors of demand that are not included in this model, such as prices. As incomes increase, income elasticities decrease. The average income elasticity for all fish species among high income countries is 0.4^2^, and the estimated global income elasticity for food in the aggregate is 0.48^91^.

Projected consumption per capita is further capped in our analysis at 37 kg/year in four countries to reflect the upper range of 100 g/day of fish consumption used in the EAT-Lancet Commission^40^. The cap is reached in China in 2026, in France in 2031, in Peru in 2039 and in Spain in 2040. Without a cap, projected per capita consumption in China would reach 56 kg/yr in edible weight in 2050.

Our model independently predicts demand for the seven categories of species-groups in live weight out to 2050. Population estimates and edible weight conversions are then used to present the per capita and edible weight predictions.

## Supplementary information

Supplementary Information

Description of Additional Supplementary Files

Supplementary Data 1

Supplementary Data 2

## Data Availability

The raw data used in this research and the data generated in this study have both been deposited in a Zenodo database^92^. There are two files that summarize these data: those related to any modeling pipelines can be found in the data folder at “bfa_demand_model_data.xlsx”; and the data associated with the creation of the figures, including data output from modeling pipelines, can be found in the data folder at “bfa_demand_figure_data.xlsx”.

## Code availability

All data analysis was performed by the authors using our own code. All code used for the analysis resulting in the figures is available on Zenodo repository^92^.
