# Author Correction: Blue food demand across geographic and temporal scales

**DOI:** 10.1038/s41467-021-26063-8

**Published:** 2021-09-28

**Authors:** Rosamond L. Naylor, Avinash Kishore, U. Rashid Sumaila, Ibrahim Issifu, Blaire P. Hunter, Ben Belton, Simon R. Bush, Ling Cao, Stefan Gelcich, Jessica A. Gephart, Christopher D. Golden, Malin Jonell, J. Zachary Koehn, David C. Little, Shakuntala H. Thilsted, Michelle Tigchelaar, Beatrice Crona

**Affiliations:** 1 Stanford University, Stanford, CA USA; 2 International Food Policy Research Institute (IFPRI), New Delhi, India; 3 University of British Columbia, Vancouver, BC Canada; 4 WorldFish, Bayan Lepas, Malaysia; 5 Michigan State University, East Lansing, MI USA; 6 Wageningen University, Wageningen, The Netherlands; 7 Shanghai Jiao Tong University, Shanghai, China; 8 Pontificia Universidad Católica de Chile, Santiago, Chile; 9 American University, Washington, DC USA; 10 Harvard T.H. Chan School of Public Health, Boston, MA USA; 11 Beijer Institute of Ecological Economics, The Royal Swedish Academy of Sciences, Stockholm, Sweden; 12 Stockholm Resilience Centre, Stockholm University, Stockholm, Sweden; 13 Royal Swedish Academy of Science, Stockholm, Sweden; 14 University of Stirling, Stirling, UK

**Keywords:** Environmental social sciences, Agriculture, Social sciences

Correction to: *Nature Communications* 10.1038/s41467-021-25516-4, published online 15 September 2021.

The original version of this Article contained errors in the author affiliations.

The affiliation of Malin Jonell and Beatrice Crona with Stockholm Resilience Centre, Stockholm University, Stockholm, Sweden was inadvertently omitted.

The affiliation of Malin Jonell with Beijer Institute of Ecological Economics, The Royal Swedish Academy of Sciences, Stockholm, Sweden was inadvertently omitted.

This has now been corrected in both the PDF and HTML versions of the Article.

