# Supplementary material for: Blue food demand across geographic and temporal scales
Source: Nat Commun. 2021 Sep 15;12:5413. doi: 10.1038/s41467-021-25516-4 (PMC8443621; doi:10.1038/s41467-021-25516-4)
Supplement: Supplementary file 1 — Supplementary Information [file 41467_2021_25516_MOESM1_ESM.pdf]

SUPPLEMENTARY INFORMATION

Blue Food Demand Across Geographic and Temporal Scales

Rosamond L. Naylor, Avinash Kishore, U. Rashid Sumaila (lead authors)
Ibrahim Issifu, Blaire P. Hunter, Ben Belton, Simon R. Bush, Ling Cao, Stefan Gelcich, Jessica A. Gephart, Christopher D. Golden, Malin Jonell, J. Zachary Koehn, David C. Little, Shakuntala H. Thilsted, Michelle Tigchelaar, Beatrice Crona

Supplemental Table 1: Highest Fish/Seafood per Capita Consumption by Country, 2017

**Country**	**2017 live weight kg/year per capita**	**2017 edible weight kg/year per capita**
Iceland	92.5	68.5
Maldives	90.5	67.0
Faroe Island	87.8	65.0
Greenland	85.7	63.4
St Pier Mq	80	59.2
Kiribati	76.7	56.8
Palau	66.8	49.4
Tokelau	66.2	49.0
Seychelles	57.9	42.8
Portugal	56.8	42.0

The countries with the highest per capita consumption of "Fish/Seafood" as food in 2017 estimated by the FAO Food Balance Sheets. Consumption is reported in live weight and converted to edible weight using a 0.74 conversion factor (see Methods). Data source: FAO. New Food Balances. (2020). Accessed April 25, 2021.

Supplemental Table 2: Species groups included in the analysis of blue food demand.

**Species Groups**	**Species**
Bivalves	Freshwater molluscs, abalones, winkles, conchs, oysters, mussels, scallops, pectens, clams, cockles, arkshells, and miscellaneous marine molluscs.
Cephalopods	Squids, cuttlefishes, and octopuses.
Crustaceans	Freshwater crustaceans, sea-spiders, crabs, lobsters, spiny-rock lobsters, squat-lobsters, shrimps, prawns, krill, planktonic crustaceans, and miscellaneous marine crustaceans
Marine Fish, Other	Miscellaneous marine fishes.
Pelagic Fish	Jacks, mullets, sauries, herrings, sardines, anchovies, tunas, bonitos, billfishes, mackerels, snoeks, and cutlassfishes.
Demersal Fish	Flounders, halibuts, soles, cods, hakes, haddocks, redfishes, basses, congers, sharks, rays, and chimaeras.
Freshwater	Carps, barbels and other cyprinids, tilapias and other cichlids, miscellaneous freshwater fishes, sturgeons, paddlefishes, river eels, salmons, trouts, smelts, shads, and miscellaneous diadromous fishes.

Categories defned by United Nations Food and Agriculture Organization: FAO. *Groups of Species*, Available at <http://www.fao.org/waicent/faostat/agricult/fishitems-e-e.html >. Accessed January 12, 2021.

Supplemental Table 3. Relative change in purchasing power parity (PPP) of Meat to Fish & Seafood, summarized for countries in our regional dataset in 2011 and 2017 using World Bank data from the International Comparison Program (ICP). PPP values indicate how much would have to be spent in local currency to purchase an equal volume of product; for all countries these volumes are calculated relative to what would be purchased by USD $1.00 (thus U.S. values are all 1.00).The two food categories are aggregated to the ICP 'basic level', and are calculated from products that are part of the global core (26 meat & 18 fish products) and regional (77 meat & 64 fish products) product lists. ICP calculates the PPP for each individual country before the basic-level calculation. All price data used in these calculations are validated by the ICP at intra-country, regional, and global levels.

	**Purchasing Power Parity**		
	**2011**	**2017**	**% change**
**Brazil**			
Meat	1.55	1.93	1.25
Fish & seafood	1.42	2.35	1.65
**China**			
Meat	5.23	5.30	1.01
Fish & seafood	3.80	5.32	1.40
**France**			
Meat	1.34	1.13	0.84
Fish & seafood	0.76	1.00	1.31
**Ghana**			
Meat	1.57	2.49	1.59
Fish & seafood	0.85	2.84	3.34
**India**			
Meat	25.88	33.91	1.31
Fish & seafood	14.91	27.56	1.85
**Mexico**			
Meat	10.35	12.14	1.17
Fish & seafood	9.37	14.05	1.50
**Nigeria**			
Meat	142.99	151.94	1.06
Fish & seafood	81.84	149.79	1.83
**Peru**			
Meat	2.00	2.16	1.08
Fish & seafood	1.73	2.56	1.48
**Spain**			
Meat	0.94	0.77	0.82
Fish and seafood	0.62	0.75	1.22
**United States**			
Meat	1.00	1.00	1.00
Fish and seafood	1.00	1.00	1.00

Data source: World Bank. Purchasing Power Parities and the Size of World Economies: Results from the 2017 International Comparison Program. Washington, DC: World Bank (2020). Doi: 10.1596/978-1-4648-1530-0. License: Creative Commons Attribution CC BY 3.0 IGO.

Supplemental Table 4a: Blue Food Consumption Values in Live and Edible Weight, 2015 and 2050

Country	2015 live weight, 1000 tonnes	2015 live weight kg/year per capita	2015 edible weight, 1000 tonnes	2015 edible weight kg/year per capita	2050 live weight, 1000 tonnes	2050 live weight kg/year per capita	2050 edible weight, 1000 tonnes	2050 edible weight kg/year per capita	2015 population, in millions	2050 population, in millions
China	53918	39	34376	25	92294	65	53027	37	1371	1359
India	8406	6	7051	5	24601	15	19835	12	1310	1639
Ghana	688	25	608	22	1912	37	1660	32	28	52
Nigeria	2035	11	1775	10	5359	13	4614	11	181	401
Peru	764	25	607	20	1889	49	1437	37	30	40
Brazil	1845	9	1516	7	5460	24	4344	19	204	229
United States	7034	22	4548	16	10423	28	6521	17	321	376
Mexico	1814	15	1361	11	6061	39	4201	27	122	155
Spain	2034	44	1398	30	2516	56	1675	37	46	43
France	2160	32	1499	23	4113	60	2622	38	67	69
Total	80698		54739		154627		99936		3681	4364

Apparent consumption is from the "Food" category from the FAO Food Balance Sheets[1] and represents seven species groups that are considered "Fish/Seafood". Population estimates are from the World Bank[2]. 2050 consumption values are predicted using the modelling described in the Methods section of the main manuscript. Assumptions and sources for key parameters are shown in Supplemental Table 4b. Edible weights are calculated by converting each species group into edible weight before being aggregated (see Methods). The consumption predictions in China, Spain, France, and Peru are capped in the first year when edible consumption per capita exceeds 37 kg, which results in a cap at the 2026, 2040, 2031 and 2039 levels, respectively. This cap is informed by the 100 g/day (36.5 kg/year) upper range of fish consumption suggested in the EAT-Lancet Commission[3]. The capped consumption values are assumed to remain the same for the remaining years of the model.

Supplemental Table 4b: Projection Model Assumptions for Key Parameters

**Parameter**	**Model Assumptions**	**Data Sources**
Population Growth	N/A	World Bank Group, 2020.
Income Growth	**2015 – 2021:** April 2020 IMF World Economic Outlook Database **2022-2024:** October 2019 IMF World Economic Outlook Database **2025-2050:** PwC The World in 2050 average annual GDP PPP growth predictions adjusted to per capita with World Bank population estimates. Ghana and Peru are not included in the PwC report. Instead, the authors estimated growth rates based on similar countries in the report and recent historical growth rates of GDP PPP per capita.	IMF, 2019. IMF, 2020. PwC, 2017. World Bank Group, Population Estimates and Projections, 2020. World Bank Group, GDP PPP per capita, 2020.
Income Elasticities	Cai and Leung 2017 species-specific income elasticity projections decrease to 0.4 by 2050 in equal annual increments or remain the same if the starting value is below 0.4.	Cai and Leung, 2017.

Data sources:

1. World Bank Group. Population Estimates and Projections. <https://datacatalog.worldbank.org/dataset/population-estimates-and-projections>. Accessed July 1, 2020.
2. IMF. *World Economic Outlook*, Available at <https://www.imf.org/en/Publications/WEO/Issues/2020/04/14/World-Economic-Outlook-April-2020-The-Great-Lockdown-49306> (April 2020). Accessed on August 10, 2020.
3. IMF. *World Economic Outlook*, Available at <https://www.imf.org/en/Publications/WEO/Issues/2019/10/01/world-economic-outlook-october-2019> (October 2019). Accessed on August 10, 2020.
4. PwC. *Global Economy Watch Projections*, Available at <https://www.pwc.com/gx/en/research-insights/economy/global-economy-watch/projections.html> (February 2017). Accessed on August 10, 2020.
5. World Bank Group. GDP per capita, PPP (constant 2017 international $). World Development Indicators (WDI), Data Catalog. <https://data.worldbank.org/indicator/NY.GDP.PCAP.PP.KD> (2019). Accessed December 17, 2020.
6. Cai, J. & Leung, P. S. *Short-term projection of global fish demand and supply gaps*. FAO Fisheries and Aquaculture Technical Paper No. 607, Rome. (2017).
7. Muhammad, A., Seale, J. L., Meade, B. & Regmi, A. *International Evidence on Food Consumption Patterns: An Update Using 2005 International Comparison Program Data*. Report No. 1929, USDA-ERS Technical Bulletin. (2011).
8. Beddington, S. J. The future of food and farming. *International Journal of Agricultural Management*. 1.2, 2-6 (2011).

Supplemental Figure 1: Global prices (nominal) for fish and terrestrial meat

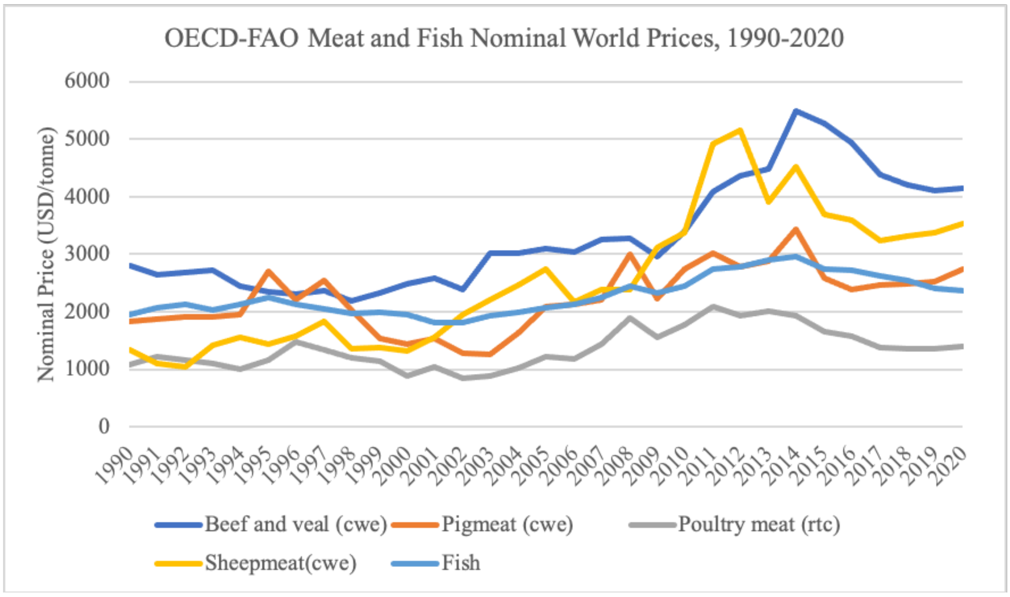

Supplemental Fig. 1a. Meat and fish nominal world prices for 1990-2020 from the OECD-FAO Agricultural Outlook (2016-2025). The fish price includes salmon and shrimp for human consumption. Prices are all USD/tonne and prices for 2015-2020 are estimates. Source: OECD-FAO Agricultural Outlook (2016-2025). The metrics "cwe" and "rtc" represent "carcass weight equivalent" and "ready to cook". Data source: OECD-FAO. *OECD-FAO Agricultural Outlook 2016-2025.* Accessed January 10, 2021. Available at https://stats.oecd.org/index.aspx?queryid=92076

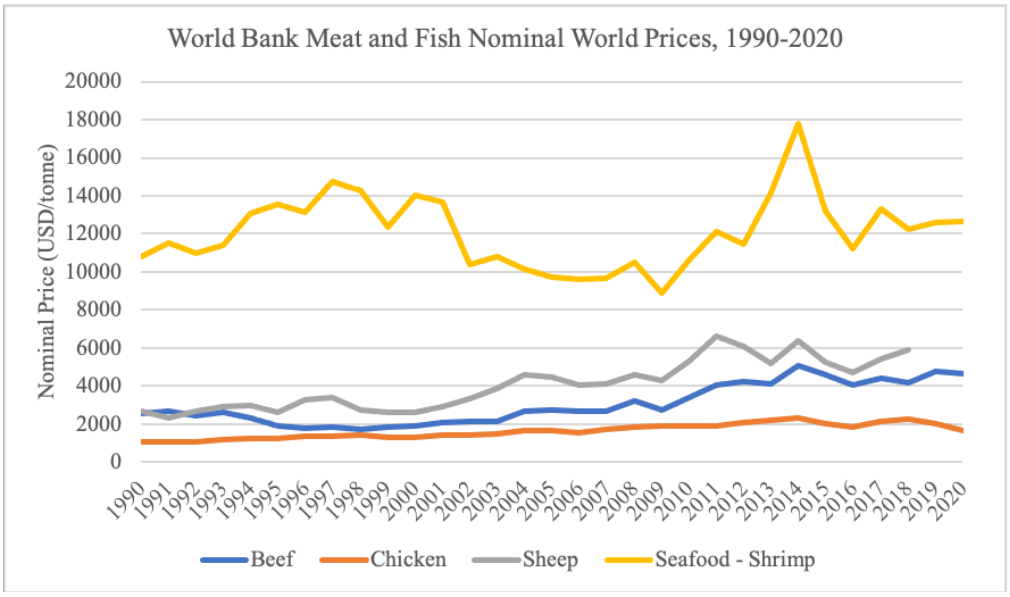

Supplemental Fig. 1b. Word Bank nominal prices for beef, chicken, sheep, and seafood-shrimp from 1960-2020. Prices are in USD/kg. The seafood prices in the world bank data are exclusively based on the internationally traded price of Mexican shrimp. Data source: World Bank Group. *Commodity Markets: Annual prices*. Accessed January 7, 2021. Available at <https://www.worldbank.org/en/research/commodity-markets>.

Prices for terrestrial animal source foods and fish (Supplemental Figures 1a and 1b) are shown in nominal values to highlight the difference in relative fish prices between the two series, and to avoid confusion in this comparison with deflator values. Price series from the OECD-FAO Agricultural Outlook (2016-2025) and the World Bank Commodity Markets convey different results pertaining to relative prices of terrestrial meat and fish. For each series, the global prices available for the seafood category are highly dependent on species included in the price category. The only marine fish available in the World Bank commodity data is Mexican shrimp, which is relatively expensive. The OECD-FAO fish series also includes high market-value fish (salmon and shrimp). Neither series includes the vast array of low market-valued fish species. As emphasized in the manuscript, fish are comprised of a much more diverse group of species than beef, pork, poultry, or sheep, which makes global price indices for fish highly dependent on how many and which species are included in the indices.

While the FAO Fish Price index, as described in Tveteras et al. (2012)[4], provides an index composed of a more diverse set of species produced by capture fisheries and aquaculture, it is derived from trade data from the EU, Japan, and the U.S. and is only a proxy for the wide variety of low-market valued species produced and consumed locally in the Global South. Moreover, we have been unable to locate the raw data that comprises the Fish Price Index update published in GLOBEFISH in order to create a relative price index for fish and terrestrial meat that matches the 30-year time period for the other indices shown in Supplementary Figures1a and 1b. Summary data for the FAO Fish Price Index and Meat Price Index are provided in the FAO 2020 State of Fisheries and Aquaculture Report for the recent decade 2010-2020[5].

References:

1 FAO. New Food Balances. (2020). Accessed August 10, 2020.

2 World Bank Group. Population Estimates and Projections. Accessed July 1, 2020. <https://datacatalog.worldbank.org/dataset/population-estimates-and-projections>.

3 Willett, W. *et al.* Food in the Anthropocene: the EAT–Lancet Commission on healthy diets from sustainable food systems. *The Lancet Commissions* **393**, 447-492, (2019).

4 Tveterås, Sigbjørn, et al. Fish is food-the FAO's fish price index. *PLoS One* 7.5 (2012).

5 FAO SOFIA. *The State of World Fisheries and Aquaculture 2020. Sustainability in action*. Rome, (2020). https://doi.org/10.4060/ca9229en.
