# Supplementary material for: Blue food demand across geographic and temporal scales
Source: Nat Commun. 2021 Sep 15;12:5413. doi: 10.1038/s41467-021-25516-4 (PMC8443621; doi:10.1038/s41467-021-25516-4)
Supplement: Supplementary file 2 — Description of Additional Supplementary Files [file 41467_2021_25516_MOESM2_ESM.pdf]

Description of Additional Supplementary Files

File Name: Supplementary Data 1
Description: Data associated with the creation of the figures in the article, including data output from modeling pipelines

File Name: Supplementary Data 2
Description: Data related to any modeling pipelines, including projected blue food demand to 2050.
